# Development and diversity of lignin patterns

**DOI:** 10.1093/plphys/kiac261

**Published:** 2022-06-01

**Authors:** Aurélia Emonet, Angela Hay

**Affiliations:** Max Planck Institute for Plant Breeding Research, Cologne, North Rhine-Westphalia, 50829, Germany; Max Planck Institute for Plant Breeding Research, Cologne, North Rhine-Westphalia, 50829, Germany

## Abstract

Different patterns of lignified cell walls are associated with diverse functions in a variety of plant tissues. These functions rely on the stiffness and hydrophobicity that lignin polymers impart to the cell wall. The precise pattern of subcellular lignin deposition is critical for the structure–function relationship in each lignified cell type. Here, we describe the role of xylem vessels as water pipes, Casparian strips as apoplastic barriers, and the role of asymmetrically lignified endocarp *b* cells in exploding seed pods. We highlight similarities and differences in the genetic mechanisms underpinning local lignin deposition in these diverse cell types. By bringing together examples from different developmental contexts and different plant species, we propose that comparative approaches can benefit our understanding of lignin patterning mechanisms.

## Teaser

Diverse lignin patterns underpin distinct functions in different plant tissues.

## Introduction

Back in the Silurian, some 400 million years ago, the world was very different from the one we know now. Plants had just started to colonize the land, developing characteristics that enabled them to survive in a dry, CO_2_-rich environment. Bryophytes, including mosses, liverworts, and hornworts, dominated the landscape (see Box 1). However, these plants were still highly dependent on water and humid conditions for their survival and reproduction. In the early Devonian, the evolution of a stiff, hydrophobic lignin polymer to reinforce plant cell walls was a game-changer. This allowed plants to develop a lignified vascular system to transport water far above the ground. Taking advantage of the newly acquired rigidity provided by lignin, vascular plants could grow taller and colonize novel ecological niches (Weng and Chapple, 2010). Although there is evidence of lignin-like material in algal lineages, it is the presence of a lignified xylem tissue that defines vascular plants (Martone et al., 2009; Sørensen et al., 2011). Moreover, xylem is not the only lignified tissue. A diverse array of cell types rely on highly localized patterns of lignin impregnation to provide specific functions and mechanics in different plants.

ADVANCES

Lignin is an aromatic polymer of monolignols derived from the phenylpropanoid pathway. Monolignol biosynthesis has been extensively studied and reviewed elsewhere (Bonawitz and Chapple, 2010; Dixon and Barros, 2019). Once exported to the apoplast, monolignols are locally activated into radicals by laccase and peroxidase oxidative enzymes, and form the lignin polymer by random coupling in the cell wall. Cell walls are usually made up of cellulose, hemicellulose, and pectin, but the addition of lignin can change their properties (Cosgrove, 1997; Boerjan et al., 2003). Specifically, lignin impregnation confers mechanical strength, rigidity, and hydrophobicity (Gibson, 2012; Dixon and Barros, 2019). Lignin is particularly resistant to degradation, to the point where a whole field of research is devoted to solve this problem for the biofuel industry (Li et al., 2008; Weng et al., 2008).

- Precise patterns of local lignin deposition have important functions in diverse tissue contexts.
- Mechanisms of local lignin deposition have a distinct genetic basis in different cell types.
- Requirement of peroxidases versus laccases for lignin polymerization differs according to developmental context.
- Innovations in lignin patterning drove phenotypic divergence across different evolutionary time scales.

**Figure 1:**
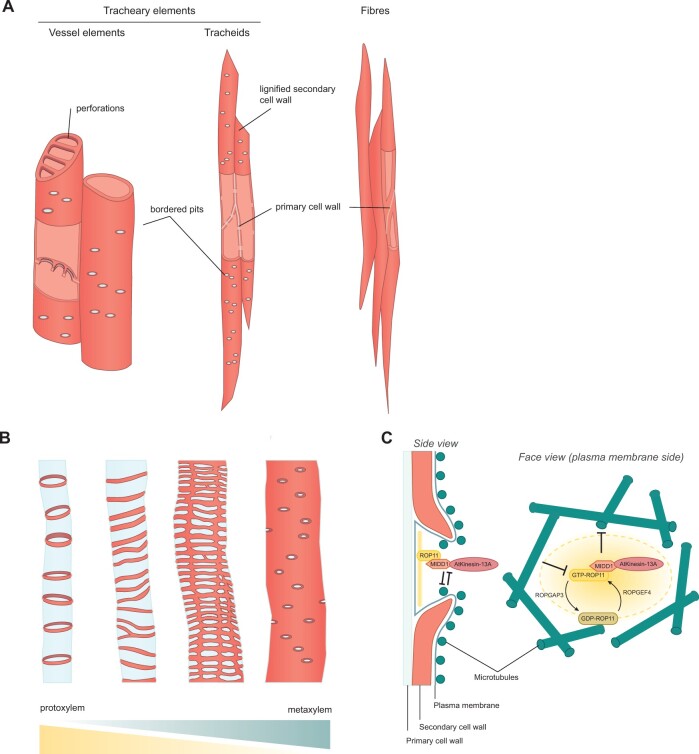
Lignin patterns in xylem cells provide rigidity and water transport across the plant. A, Xylem tissues comprise several lignified cell types: vessels elements and tracheids, which are tracheary elements that transport water and fibers. B, Lignin patterns (red) observed in primary xylem tissues: from left to right, annular, spiraled, reticulated-scalariform, pitted. Protoxylem cells tend to have annular or spiraled patterns, while metaxylem cells are mostly pitted. Primary cell wall shown in blue. C, Mechanism of bordered pit formation in Arabidopsis metaxylem viewed from the side (left) and facing the plasma membrane (right). Activated ROP11 forms islands in the plasma membrane by a process that could be explained by Turing’s reaction diffusion mechanism (Oda and Fukuda, 2012). Activated ROP11 recruits MIDD1 and AtKinesin-13A proteins to depolymerise microtubule ends. Microtubules restrict expansion of ROP11 islands in the membrane. Figures inspired from Oda and Fukuda (2013).

**Figure 2:**
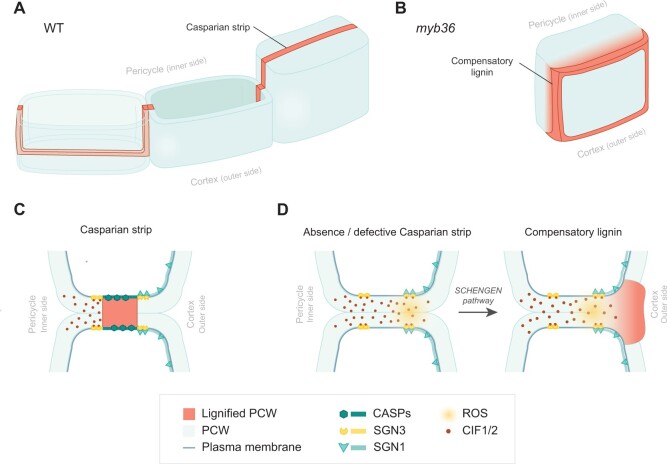
Casparian strips and compensatory lignin—two ways to seal the apoplast. A, Casparian strips are localized lignin impregnations (red) of the primary cell wall (blue), forming a band that surrounds each endodermal cell and seals the apoplast. Cartoon shows three adjacent cells: from left to right, transparent view of cut cell, nontransparent views of cut and uncut cells showing Casparian strip in surface view. B, Compensatory lignin (red) deposited in the primary cell wall (blue) in endodermal cell corners in response to defects or absence of Casparian strips (e.g. *myb36* mutant). C, Casparian strip formation relies on the precise localization of CASP proteins that are likely to act as a scaffold for lignin polymerizing enzymes. When completed, the Casparian strip blocks the diffusion of CIF1/2 peptides. Cartoon shows Casparian strip in median view. D, When the Casparian strip is defective or absent (e.g. *myb36* mutant), CIF1/2 can diffuse through the apoplast and reach the SGN3–SGN1 complex. This triggers ROS production necessary for monolignol activation by peroxidases, and subsequent compensatory lignin polymerization. PCW, primary cell wall; CASP, CASPARIAN STRIP DOMAIN PROTEIN; SGN, SHENGEN; CIF, CASPARIAN STRIP INTEGRITY FACTOR.

**Figure 3:**
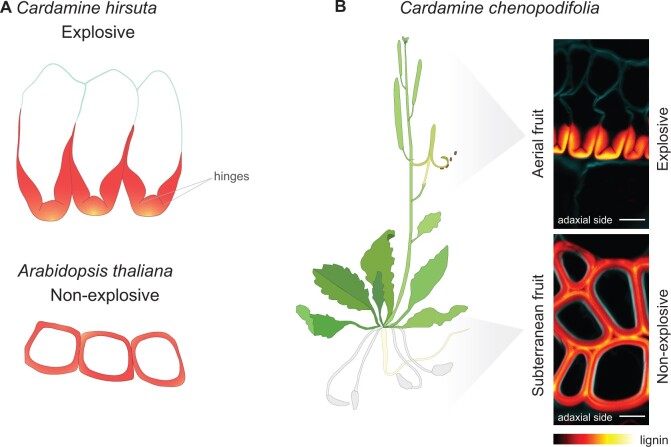
Polar pattern of lignin deposition in endocarp *b* cells of explosive fruit. A, Lignin pattern (red) in endocarp *b* cells of explosive *C. hirsuta* fruit and non-explosive Arabidopsis fruit. In *C. hirsuta*, lignin is deposited asymmetrically, forming a “U”-shape in cross section, with thinner hinges embedded in the thick wall. Nonlignified cell wall shown in blue. In Arabidopsis, lignin is deposited symmetrically. B, The amphicarpic species *C. chenopodifolia* has explosive aerial fruit and non-explosive subterranean fruit. In *C. chenopodifolia* endocarp *b* cells, lignin is deposited asymmetrically in explosive fruit, in a similar pattern to *C. hirsuta*, and symmetrically in non-explosive fruit, in a similar pattern to Arabidopsis. Confocal micrographs of endocarp *b* cells show lignin stained with Basic Fuchsin (RedHot LUT) and cellulose with Calcofluor White (cyan). Adaxial side of the valve is indicated. Scale bar: 10 μm. Imaging performed on a Leica SP8 confocal scanning microscope, 63× water immersion objective lambda-blue. Excitation and detection windows: for Basic Fuchsin (561 nm, 600–650 nm), for Calcufluor White (405 nm, 410–520 nm). Data from A.E.

The functions conferred by lignin depend not only on its presence in the cell wall, but also on the spatial patterns of lignin deposition. In this review, we describe examples of spiraled or pitted patterns in xylem vessels (Figure 1), net-like structures of Casparian strips in the endodermis (Figure 2), and asymmetric, “U”-shaped depositions in cells of explosive *Cardamine* fruit (Figure 3). These intricate and varied structures can either be very conserved across vascular plants; for example, tracheary elements or Casparian strips, or associated with lineage-specific traits, such as explosive fruit of the *Cardamine* genus. This review aims to link the structures formed by lignin patterns in the cell wall to specific functions, and to bridge the gap between diverse lignin patterns and their genetic underpinnings.

**Box 2 figure:**
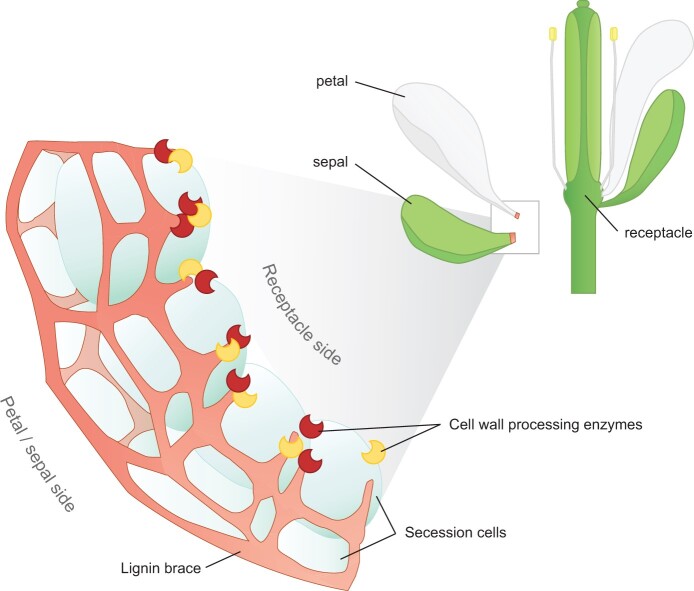
Honeycomb lignin structure in Arabidopsis petal and sepal abscission zones. During floral organ abscission, petals and sepals detach from the flower at the receptacle. Cells at the boundary of petals and sepals, called secession cells (blue), develop a honeycomb structure of lignin (red) at their corners. This lignin structure acts as a brace to keep secession cells together and forms an apoplastic barrier to limit the diffusion of cell wall processing enzymes (dark red and yellow crescents) in the abscission zone. Figure inspired from Lee et al. (2018).

## Lignin for water pipes: xylem

Although nonvascular plants have water conducting cells (see Box 1), lignified xylem tissue is an innovation of vascular plants (Esau, 1960). These highly lignified, hollow cells are ideal structures to fit end to end and function as water pipes. The development of an efficient vascular system allowed plants to transport water over vertical distances greater than 100 m in the tallest trees, which was not possible with the hydroids found in bryophytes (Brodribb et al., 2020). Therefore, xylem tissue provided two vital functions. First, it allowed the transport of water and nutrients from the root to the shoot, driven by the negative hydraulic pressure generated by leaf transpiration. Second, it provided the mechanical support necessary for plants to grow tall and extend their photosynthetic leaves far above the soil surface (Esau, 1960).

Xylem tissues are composed of several cell types, the most characteristic being tracheary elements and fibers (Figure 1A). Tracheary elements function in water conductance and support, while fibers specifically function in mechanical support. The massive secondary cell wall of fibers is usually plain or sometimes punctuated by small pits. In contrast, tracheary elements enable the passage of water and are classed in two types depending on the presence of perforations in their cell wall. Long, thin tracheids, found in all vascular plants, are nonperforated, but allow the flow of water to pass through small pits, that is, holes in the secondary cell wall where the remnant primary cell wall is highly permeable. Vessel elements, found only in angiosperms, are larger, hollow cells with perforations at each end, together with bordered pits on their side walls (Figure 1A). Therefore, the specialization of vessels and fibers in Angiosperms separates the two functions of water transport and structural support that are performed only by tracheids in most Gymnosperms (Bailey, 1953).

Tracheary elements rely on a lignified secondary cell wall for hydrophobicity—to channel water through thin pipes, flowing from one cell to the other through the perforations and pits patterned in the wall—and for strength—to resist the extreme negative hydraulic pressure induced by transpiration and to avoid implosion, also called transverse buckling (Sperry, 2003; Stroock et al., 2014). Despite providing the same mechanical functions, the lignified secondary cell walls of tracheary elements can show a variety of different patterns in primary xylem (Figure 1B). This xylem tissue is derived from the procambium during primary growth and differs from secondary xylem, which is derived from the vascular cambium after the transition to secondary growth (Esau, 1960). Protoxylem cells in the primary xylem present a delicate helical pattern, while metaxylem cells are mostly covered with lignified secondary cell walls except for the pits. In-between these two extremes, a range of lignin patterns can be observed, from annular, reticulated to scalariform (Figure 1B; Esau, 1960; Schuetz et al., 2013; Růžička et al., 2015). Protoxylem cells differentiate in the context of growing tissues and face the problem that lignification restricts cell growth. A helical lignin pattern solves this problem by functioning like the hosepipe of a vacuum cleaner—allowing flexibility and continuous axial elongation while providing resistance. In contrast, metaxylem cells differentiate later in development after the plant body has elongated. Since flexibility is no longer required, these cells tend to have pitted or reticulate patterns of lignin (Figure 1B; Esau, 1960; Růžička et al., 2015) .

The genetic regulation of these intricate lignin patterns has been the focus of much work, including studies that leverage the power of the model species Arabidopsis (*Arabidopsis thaliana*; Rogers and Campbell, 2004; Schuetz et al., 2013; Nakano et al., 2015; Rao and Dixon, 2018). Several master regulators of xylem differentiation have been identified: NST1 (NAC SECONDARY WALL THICKENING PROMOTING FACTOR 1) and NST3/SND1 (SECONDARY WALL-ASSOCIATED NAC DOMAIN PROTEIN 1) were shown to control fiber differentiation, while VND6 and 7 (VASCULAR-RELATED NAC-DOMAIN) drive metaxylem and protoxylem formation, respectively (Kubo et al., 2005; Mitsuda et al., 2007; Yamaguchi et al., 2008, 2011; Schürholz et al., 2018). Ectopic expression of VND6 and VND7 are sufficient to induce pitted and helical patterns of lignified secondary cell walls, respectively, in nonxylem cell types (Kubo et al., 2005; Yamaguchi et al., 2008; Schürholz et al., 2018). These transcription factors control a complex gene regulatory network that is discussed elsewhere (Nakano et al., 2015; Růžička et al., 2015; Taylor-Teeples et al., 2015). Exactly how these master regulators determine different lignin patterns is not yet clear, but much progress has been made towards understanding the mechanisms that regulate local lignin deposition.

Cortical microtubules play a key role in this process. Their local accumulation at the cell cortex determines the trajectories along which cellulose synthase complexes at the plasma membrane extrude cellulose microfibrils into the secondary cell wall (Hepler and Newcomb, 1964; Paredez et al., 2006; Wightman and Turner, 2008; Watanabe et al., 2015). Pits form in the lignified secondary cell walls of metaxylem cells via the depolymerization of cortical microtubules, which consequently prevents cellulose synthesis and lignin deposition in pit regions (Figure 1C). Specifically, activated RHO-RELATED PROTEIN FROM PLANTS 11 (ROP11) localizes to the plasma membrane at pit sites, and recruits MICROTUBULE-DEPLETION DOMAIN 1 (MIDD1) and kinesin-13A in order to depolymerize cortical microtubules (Figure 1C; Oda et al., 2010; Oda and Fukuda, 2012, 2013). The localization of activated ROP11 is likely to be patterned by a reaction-diffusion mechanism involving the opposite actions of the activator ROP GUANINE NUCLEOTIDE EXCHANGE FACTOR 4 (ROPGEF4) and the inhibitor ROP GUANOSINE TRIPHOSPHATASE-ACTIVATING PROTEIN 3 (ROPGAP3); Figure 1C; Oda and Fukuda, 2012; Nagashima et al., 2018). The shape of the active ROP11 domains is likely to be maintained by feedback from microtubules, microtubule-associated proteins, and actin signaling (Sasaki et al., 2017; Sugiyama et al., 2017, 2019; Nagashima et al., 2018). However, a different mechanism may regulate helical patterning of lignified secondary cell walls, since mutations in many of the genes described above have only weak phenotypes in protoxylem (Schneider et al., 2021).

Secondary cell wall patterns are lignified through targeted polymerization of monolignols. The monolignols that are required for fiber and vessel lignification are produced cell autonomously for interfascicular fibers, but also noncell autonomously for vessel elements. Following the good-neighbor hypothesis, vessel elements can receive monolignols from adjacent xylary parenchyma cells or ray cells in order to lignify post-mortem (Pesquet et al., 2013; Smith et al., 2013, 2017). It is not yet clear how monolignols move from the cytoplasm to the cell wall, as active transport and passive diffusion are both described as possibilities (Miao and Liu, 2010; Alejandro et al., 2012; Perkins et al., 2019; Vermaas et al., 2019). Once they reach the apoplast, these mobile monomers are locally polymerized into lignin via oxidation by laccases and peroxidases and subsequent radical coupling (Meents et al., 2018). Recent work shows how this polymerization can generate a concentration gradient to drive passive diffusion of monolignols from the cytoplasm to the cell wall (Perkins et al., 2022).

Laccases and peroxidases are secreted glycoproteins that play an important role in determining where and when lignin is locally polymerized. Laccases seem to have a predominant role in vessel elements since *lac4 11 17* triple mutants have severe growth phenotypes and strongly reduced stem lignification (Berthet et al., 2011; Zhao et al., 2013). However, specific laccases and peroxidases might function in different parts of the cell wall (Hoffmann et al., 2020). For example, while LAC4 (LACCASE), LAC17, and PER72 (PEROXIDASE) are embedded in the secondary cell wall of xylem vessels and fibers, PER64 and PER71, and transiently LAC4, localize to the cell corners and middle lamellae of fibers at the onset of lignification (Schuetz et al., 2014; Chou et al., 2018; Hoffmann et al., 2020). Interestingly, different laccases might not only determine where lignin is polymerized in the cell wall, but also which types of monolignols are integrated into lignin, as observed in gymnosperm compression wood (Hiraide et al., 2021). Exactly how laccases and peroxidases are targeted to specific cell wall domains is still unclear. Evidence suggests that cortical microtubules might guide the secretion of these oxidative enzymes and cell wall polysaccharides independently of cellulose during protoxylem development (Chou et al., 2018; Meents et al., 2018; Takenaka et al., 2018). Once secreted, oxidative enzymes could also be immobilized in the secondary cell wall matrix, since LAC4 showed reduced mobility in secondary compared to primary cell walls (Chou et al., 2018). Therefore, bridging the gap between microtubules and the lignin polymerizing machinery will be an important step towards understanding lignin pattern formation in xylem cells.

## Lignin for apoplastic barriers: Casparian strips and compensatory lignin

While xylem relies on the strength and hydrophobicity of lignin to form rigid tubes carrying water across the plant, other tissues use these same properties to create tight barriers between cells (see Box 2). The lignified Casparian strips provide this barrier function in the root endodermis. The endodermis surrounds the root vasculature and regulates what can go in and what should stay out. Importantly, it also ensures that what enters the root does not leak out following its natural gradient. To effectively control ion homeostasis, the direct apoplastic route for charged ions to diffuse between cells is blocked by lignified Casparian strips, so that ion fluxes are redirected towards endodermal cells following the symplastic or the coupled trans-cellular pathway (i.e. the successive import and export of ions across plasma membranes; Enstone et al., 2002; Naseer et al., 2012; Pfister et al., 2014; Barberon, 2016; Barberon et al., 2016). This apoplastic block, caused by lignified Casparian strips, is independent of suberin lamellae that appear later in endodermal development and block the coupled trans-cellular pathway for ion transport (Naseer et al., 2012; Barberon et al., 2016). Casparian strip mutants with a fully permeable root show major shoot ionomic changes, severe growth defects and reduced fitness, illustrating the importance of the apoplastic barrier for the bi-directional control of ion homeostasis (Pfister et al., 2014; Kamiya et al., 2015; Doblas et al., 2017; Reyt et al., 2021).

OUTSTANDING QUESTIONS

Unlike the xylem, Casparian strips are not lignified secondary cell wall depositions, but are formed by a local impregnation of lignin in the primary cell wall of living endodermal cells, spreading across the middle lamellae between cells. Casparian strips are tightly associated with the plasma membrane, even after plasmolysis (Bonnett, 1968; Enstone et al., 2002; Roppolo et al., 2011; Reyt et al., 2021). Each Casparian strip is a delicate, ring-like structure of lignin, deposited along the anticlinal and transverse walls of an endodermal cell. This links all endodermal cells together and locally seals the apoplastic space (Figure 2A; Caspary, 1865; Alassimone et al., 2010; Geldner, 2013). In this way, local lignin deposition enables the endodermis to function as a gate-keeper of the absorption system of the plant.

- How are nonmodel organisms best harnessed for comparative approaches to understand lignin patterning?
- How can we identify functionally relevant patterns of laccases and peroxidases when genetic and biocatalytic redundancy is pervasive?
- How do monolignols move to the apoplast? Do mechanisms vary according to developmental context?
- What determines the localization of laccase and peroxidase enzymes in different domains of the cell wall?
- What mechanisms act in different cell types to link microtubules at the cell cortex with lignin polymerizing machinery in the cell wall?
- To what extent is the function of the SHENGEN pathway in Casparian strip formation conserved across species?
- Are analogous barrier surveillance systems used to establish other apoplastic barriers?
- To what degree is cell corner lignification similar in response to developmental versus damage response signals?

How the endodermis is able to localize lignin deposition precisely at the Casparian strip domain has been the focus of several genetic studies in Arabidopsis. CASP transmembrane proteins (CASPARIAN STRIP DOMAIN PROTEINS 1–5) localize and oligomerize at the Casparian strip domain, where they are required to scaffold the formation and fusion of the Casparian strip into a continuous band (Figure 2C; Roppolo et al., 2011, 2014). *CASP* genes are targets of the transcription factor MYELOBLASTOSIS PROTO-ONCOGENE 36 (MYB36); Roppolo et al., 2011; Kamiya et al., 2015). The positional information that targets CASPs to the Casparian strip domain is not yet clear, but involves the exocyst complex subunit EXO70A1 and the putative extracellular protease LOTR1 (Kalmbach et al., 2017; Kolbeck et al., 2022). Lignification of the Casparian strip requires monolignols, produced autonomously by the endodermis (Andersen et al., 2021), to be activated by peroxidases to form the lignin polymer. Peroxidases reduce H_2_O_2_ to oxidize monolignols, and both PER64 and NADPH-oxidase RESPIRATORY BURST OXIDASE HOMOLOG F (RBOHF), which is required for local production of reactive oxygen species (ROS), precisely co-localize with CASP proteins at the Casparian strip (Roppolo et al., 2011; Lee et al., 2013; Barbosa et al., 2019). Uclacyanin1, a copper-containing phytocyanin potentially involved in redox reactions, is also required for, and localizes to, Casparian strips (Reyt et al., 2020). Although several laccases show endodermal expression, lignification of the Casparian strip depends on peroxidases rather than laccases, as a nonuple laccase mutant (*lac1 3 5 7 8 9 12 13 16*) did not display a Casparian strip phenotype. In comparison, Casparian strips were completely abolished in a quintuple peroxidase mutant (*per3 9 39 72 64*; Rojas-Murcia et al., 2020). This is very different to xylem lignification, which mostly depends on laccases as described above (Zhao et al., 2013; Schuetz et al., 2014).

Surprisingly, characterization of various Casparian strip mutants in Arabidopsis revealed a second and distinct lignin impregnation in the endodermis. Localized in the primary cell wall at the outer cell corners of the endodermis (facing the soil), this ectopic lignification is triggered in response to defects in the Casparian strip diffusion barrier (e.g. *myb36* mutant, Figure 2B; Roppolo et al., 2011; Hosmani et al., 2013; Kamiya et al., 2015; Li et al., 2017). This so-called “compensatory” or “stress”-lignin is chemically distinct from the Casparian strip and goes hand in hand with early suberin deposition (Doblas et al., 2017; Reyt et al., 2021; Wang et al., 2019a). In contrast to the Casparian strip, stress-lignin does not depend on autonomous monolignol production (Andersen et al., 2021). Compensatory lignification is the result of an overstimulation of the SGN (SCHENGEN) pathway. This pathway functions as a surveillance system to monitor the integrity of Casparian strips and is required for the formation of a continuous Casparian strip domain during normal development (Figure 2C; Pfister et al., 2014; Alassimone et al., 2016; Doblas et al., 2017). Two small peptides CIF1 and 2 (CASPARIAN STRIP INTEGRITY FACTORS) are produced in the root stele, and, when sulfated by the SGN2 tyrosylprotein sulfotransferase, can freely diffuse through the apoplast across the root (Doblas et al., 2017; Nakayama et al., 2017; Fujita et al., 2020; Okuda et al., 2020). In the endodermis, their cognate receptor SGN3/GSO1 (GASSHO1) surrounds the Casparian strip domain (Pfister et al., 2014). However, the downstream receptor-like cytoplasmic kinase SGN1, which is required for signaling, is located only at the outer domain of the endodermis (Figure 2C; Alassimone et al., 2016; Fujita et al., 2020). Therefore, the three partners (CIF–SGN3–SGN1) can meet at the outside corners of Casparian strips only when the barrier is leaky or missing, letting CIF1/2 diffuse across the endodermis (Doblas et al., 2017). Thus, in most Casparian strip mutants, CIF1 and 2 continuously overstimulate the SGN complex, leading to local ROS production by RBOHF and RBOHD at the cell corners and subsequent oxidation and polymerization of monolignols (Figure 2D). This causes ectopic lignin deposition, which forms an alternative apoplastic barrier at the outside cell corners in an attempt to compensate for gaps in the Casparian strips (Figure 2B; Roppolo et al., 2011; Hosmani et al., 2013; Kamiya et al., 2015; Doblas et al., 2017; Li et al., 2017; Fujita et al., 2020). Thus, the SGN pathway has a dual role in the formation of two distinct but complementary types of apoplastic barriers in the root endodermis.

Whether these two different lignin structures are equally effective barriers is unclear. Stress-lignin can essentially compensate for the permeability of Casparian strip mutants, whereas the absence of both types of apoplastic barriers causes a fully permeable root (e.g. *myb36 sgn3* double mutant) (Roppolo et al., 2011; Hosmani et al., 2013; Kamiya et al., 2015; Li et al., 2017; Reyt et al., 2021). However, Casparian strip mutants with compensatory lignin still display a slight delay of the apoplastic block compared to wild type. One possible explanation is that the compensatory lignin barrier is slightly less efficient than the Casparian strips, as the lignified cell wall corners are not directly attached to the plasma membrane (Reyt et al., 2021). Although plasma membrane attachment is always observed when Casparian strips are functional, there is so far no evidence that this is strictly required, in turgid cells, for an effective apoplastic block (Alassimone et al., 2010; Roppolo et al., 2014; Reyt et al., 2021). An alternative possibility to explain the delayed apoplastic block associated with compensatory lignin, is that it simply takes longer to deposit lignin throughout the large endodermal cell corners, compared to the restricted size of the Casparian strips. Compensatory lignin would eventually form an effective apoplastic barrier, which is seen by the complete block of apoplastic tracers in the later part of the root (Roppolo et al., 2011; Hosmani et al., 2013; Kamiya et al., 2015; Li et al., 2017; Reyt et al., 2021). Future studies investigating the function of the Casparian strip attachment to the plasma membrane might help to test these hypotheses.

Although the genetic regulation of Casparian strips and compensatory lignin share common components in Arabidopsis, is it so far unclear whether the pathways that form these distinct lignin patterns are conserved across different species, and how such pathways evolved (Li et al., 2018; Wang et al., 2019b, 2020). Comparing SCHENGEN and immune response pathways might help to shed light on the relationship between developmental and immune-induced apoplastic barriers (see Box 3). Moreover, characterizing the formation of Casparian strips, compensatory lignin and immune-response lignin in different species might provide new insights into the degree of conservation and divergence between these mechanisms.

BOX 1 Water transport without lignin.

A lignified vascular system was a major innovation of vascular plants. However, nonvascular plants already possessed cell types with similar functions (Brodribb et al., 2020). Sclereids and water-conducting hydroids are specialized cell types found in many living and extinct bryophytes. Hydroids have no cellular content like tracheary elements, but their cell walls are not lignified and generally lack pits. Sclereids are functionally related to fiber cells and have thick cell walls that lack lignin (Ligrone et al., 2000). Surprisingly, these cell types are regulated in bryophytes by ancestral VND and NST/SND genes, which are master regulators of xylem differentiation in Arabidopsis. For example, *PpVNS1 (VND, NST/SND, SMB-related gene 1)*, *PpVNS6*, and *PpVNS7* regulate the development of hydroids and sclereids in the moss *Physcomitrium patens. PpVNS* gene function was astonishingly conserved between *P. patens* and Arabidopsis, and sufficient to induce ectopic lignin deposition when transformed in Arabidopsis (Xu et al., 2014). This raised the question whether water-conducting hydroids in bryophytes could provide the same function as lignified xylem tissues. The efficiency of water transport and resistance to buckling and cavitation in hydroids was recently demonstrated in the moss *Polytrichum commune* (Brodribb et al., 2020). Moreover, although *P. commune* lacks stomata, it can regulate water exchange through leaf curling. However, poor water use efficiency (i.e. the exchange ratio of water for photosynthetic CO_2_) and sensitivity to humidity probably prevented bryophytes from competing with vascular plants for ecological niches far above the ground surface (Brodribb et al., 2020). Therefore, lignified xylem and fibers were just one of a suite of innovations associated with the improved water use efficiency of vascular plants.

## Lignin for rapid movements: exploding seed pods

In contrast to the conserved functions of lignin structures found in either the xylem or the endodermis, patterns of local lignin deposition can also be associated with trait divergence, at more shallow evolutionary time scales. For example, the seed pods of *Cardamine hirsuta*, a close relative of Arabidopsis (Hay and Tsiantis, 2006; Hay et al., 2014), employ an explosive mechanism to disperse their seeds, which relies on a specific pattern of local lignin deposition. During the explosion of *C. hirsuta* seed pods, the two valves coil rapidly at speeds greater than 10 m s^−1^, launching the seeds on ballistic trajectories to spread over a large area (Hofhuis et al., 2016). This is an effective dispersal strategy for a ruderal species like *C. hirsuta* and is found in various plants, including invasive weeds like the touch-me-not *Impatiens glandulifera* (Deegan, 2012).

How do the small seed pods of *C. hirsuta* achieve such astonishing speeds? The key is a mechanical instability (Skotheim and Mahadevan, 2005) used to rapidly transform stored elastic potential energy into kinetic coiling energy. This mechanism relies on a unique pattern of polar lignin deposition in a single-cell layer of the fruit valve, called the endocarp *b* (Hofhuis et al., 2016). This fruit layer is commonly lignified; for example, forming the hard stone in peach or cherry fruit (Dardick and Callahan, 2014). Compared to the nonexplosive fruit of Arabidopsis, where endocarp *b* cells are lignified uniformly, these cells are asymmetrically lignified in *C. hirsuta* (Figure 3A). Lignin is deposited in three thick rods on the adaxial side of each endocarp *b* cell, forming a “U” shape in cross section with thin hinges connecting each rod (Figure 3A). The hinged geometry of these lignified cells allows the fruit valve to employ a rapid release mechanism like a toy slap bracelet. The curved cross-sectional geometry of the valve imposes an energetic barrier, such that tension produced by differential contraction of valve tissues generates stored elastic potential energy. Unusually, this contraction is an active process that is driven by turgor pressure, rather than by drying of the fruit tissues (Hofhuis et al., 2016). Once sufficient tension is established, the thin hinges in the lignified cell walls open, causing the valve to change from a curved to a flat cross-section (Hofhuis et al., 2016). This change in geometry overcomes the energetic barrier and triggers rapid coiling of the valve.

The relationship between the structure of the lignified endocarp *b* cell wall and its function in exploding seed pods, was studied in *C. hirsuta* using genetics and mathematical modeling. A mutant lacking the lignified endocarp *b* cell layer was isolated from a forward genetics screen in *C. hirsuta*, and found to be caused by mutation of the putative ortholog of the DNA-binding protein BRASSINOSTEROID-INSENSITIVE 4 (Hofhuis et al., 2016). This mutant has nonexplosive fruit, indicating that the lignified endocarp *b* cell layer is required for explosive coiling of the fruit valves. Mathematical modeling was used to show that it is the precise pattern of lignin in endocarp *b* cell walls that is required for explosion. Simulations of a model that described the elastic energy in the fruit valve were compared using a hinged versus a uniformly lignified wall geometry. These results showed that the hinged wall geometry is critical for explosive energy release (Hofhuis et al., 2016). Predictions from these model simulations were tested using ectopic expression of the VND7 transcription factor in *C. hirsuta* fruit valves. This created uniformly lignified endocarp *b* cells that could no longer “open” and function as a rapid energy release mechanism. These seed pods failed to explode, showing that the asymmetric pattern of lignin deposition in endocarp *b* cells is required for explosive seed dispersal (Hofhuis et al., 2016).

Recent work has started to explore the genetic regulation of localized lignin deposition in *C. hirsuta* fruit. Another mutant from the screen described above (Hofhuis and Hay, 2017) showed a reduction in endocarp *b* cell wall lignification and a consequent reduction in seed dispersal range. This phenotype is caused by the loss of *SPL7* (*SQUAMOSAPROMOTER-BINDING PROTEIN-LIKE 7*) gene function (Pérez Antón et al., 2022). The SPL7 transcription factor is a conserved regulator of copper homeostasis and is required for copper to accumulate in the fruit of *C. hirsuta*. This finding led to the discovery that endocarp *b* cell wall lignification is laccase dependent. Laccases are copper-requiring enzymes and their activity depends on the SPL7 pathway to provide sufficient copper for lignification. Specifically, *C. hirsuta* LAC4, LAC11, and LAC17 proteins perfectly co-localize with lignin in the endocarp *b* cell wall and are required for lignification (Pérez Antón et al., 2022). These are the same laccases that are required for xylem lignification in Arabidopsis (Berthet et al., 2011; Zhao et al., 2013). This laccase dependence differs from the peroxidase-dependent lignification of Casparian strips described above (Rojas-Murcia et al., 2020).

Polar deposition of the lignified endocarp *b* secondary cell wall is not only required for explosive seed dispersal in *C. hirsuta*, but evolved in striking association with the trait. Phylogenetic comparisons within the Brassicaceae, showed that this polar lignin pattern is an innovation of the *Cardamine* genus where the trait of explosive seed dispersal evolved (Hofhuis et al., 2016). In contrast, the endocarp *b* cells of other nonexplosive Brassicaceae have symmetrically lignified cell walls. Within this strict association, one *Cardamine* species stands out as an exception. *Cardamine chenopodifolia* is amphicarpic—in addition to the aerial explosive fruit found in all *Cardamine* species, it also produces subterranean fruit (Persoon, 1807). These fruits are nonexplosive and produce only a handful of large seeds. In contrast to peanut plants (*Arachis hypogaea*), that flower in the air then bury their fruit, the main stem of *C. chenopodifolia* does not bolt, and produces cleistogamous flower buds that are immediately buried into the ground by extensive growth of their long pedicels (Cheplick, 1983). These distinctive properties already inspired botanists in the 19th century to record that the different fruit types had different patterns of endocarp *b* lignification (Gorczyński, 1930). The presence of polar lignin deposition in aerial explosive fruit, and symmetric lignification in subterranean nonexplosive fruit, makes this an interesting species for comparative studies of lignin patterning (Figure 3B).

## Conclusion

The evolution of lignin, and its subsequent deployment in different tissue types of the plant body, underpinned key innovations associated with the diversification of vascular plants. The ability to precisely control the subcellular deposition of lignin was fundamental for these adaptations. Although recent advances have started to uncover the mechanisms of local lignin deposition in different cell types, the degree of conservation versus divergence between these mechanisms, and across species, is far from being understood. In this review, we discussed examples of different lignin patterns from distinct developmental contexts, in order to highlight how advances in one field of research could influence others. For example, lignin polymerizing enzymes co-localize precisely with lignin in each cell type, suggesting that this step of lignin formation is a key determinant of patterning. The relative requirement for peroxidases versus laccases, however, differs between cell types. Lignin patterning in the xylem relies on local depletion of the secondary cell wall (Figure 1C), and it will be interesting to understand whether or not the hinged patterns in endocarp *b* cell walls of explosive fruit rely on a similar mechanism. Sites of Casparian strip lignification in endodermal cells are determined by CASP protein localization (Figure 2C), and identifying factors that determine the polarity of lignin deposition in endocarp *b* cells may indicate to what extent cell-specific mechanisms determine local lignin deposition. Moreover, surveillance systems similar to the SGN pathway have been found to probe other nonlignified barriers (Creff et al., 2019; Doll et al., 2020). Identifying analogous systems in different tissues might help to understand the evolution of these pathways. Comparative studies may, therefore, be a way forward to understand how mechanisms of lignin deposition can arise and be modified or redeployed to evolve specific forms and functions (see Outstanding Questions). Current advances in long read sequencing and genome editing (CRISPR-Cas9) offer exciting opportunities to address these questions in a range of plants and will prove indispensable to understand the development and diversity of lignin patterns and functions.

BOX 2 Dual lignin functions in the floral abscission zone.

Lignin is a hallmark of abscission. This important process is required for shedding leaves in autumn, discarding floral organs after fertilization, opening dehiscent fruit, and dispersing seeds. The abscission or dehiscence zone that forms between the plant and its shedding structure, usually comprises a lignified region adjacent to a nonlignified region where cell autolysis occurs (Ballester and Ferrándiz, 2017; Lee, 2019). The general dogma that lignin seals the wound generated by the abscission process was considerably updated by a detailed study of floral organ abscission (Lee et al., 2018). Rather than uniform lignification sealing the exposed cell surface, a honeycomb structure of lignin forms a brace surrounding the secession cells on the shed organs (see figure in Box 2). This lignin structure was found to have a dual function. First, it braces the layer of separating cells together and guarantees their proper detachment from the flower receptacle. A testable hypothesis is that the lignin brace provides mechanical properties such as transverse shear stiffness. Second, the lignification of secession cell corners acts as a barrier to spatially limit the diffusion of cell wall degrading enzymes in the abscising organ. This ensures that cell wall breakdown is precisely targeted to a narrow zone of abscising cells. The function of lignin as a diffusion barrier might, therefore, be more important than previously thought for abscission processes.

BOX 3 Compensatory lignin—a defense response repurposed for development?

Lignin deposition is a classical response to pathogens (Miedes et al., 2014). However, it has only recently been demonstrated that lignification is dependent on microbe-associated molecular pattern (MAMP)-triggered and effector-triggered immune responses (MTI and ETI; Chezem et al., 2017; Lee et al., 2019; Kim et al., 2020). Stimulation of PEPR1 (PEP1 RECEPTOR) and PEPR2 receptor kinases by their ligand AtPep1 induces strong lignin deposition in the root (Engelsdorf et al., 2018). Lignification is also triggered by flagellin treatment of Arabidopsis plants that ectopically express the immune receptor FLAGELLIN SENSING 2 in different root tissues (Emonet et al., 2021). Intriguingly, SCHENGEN-dependent compensatory lignin in the endodermis shares many similarities with MTI-induced lignin. Both pathways use receptors, co-receptors, and associated kinases from the same gene families and induce MITOGEN-ACTIVATED PROTEIN KINASE (MAPK) phosphorylation cascades that activate MAPK3 and MAPK6 (Creff et al., 2019; Fujita et al., 2020; Okuda et al., 2020). In addition, both pathways induce ROS production through RBOHF and RBOHD, which is required for monolignol activation by peroxidases. The SCHENGEN ligand, CIF2, induces transcriptional changes in both defense and lignin biosynthesis genes in Arabidopsis roots (Fujita et al., 2020), including MYB15 that controls lignin deposition in response to MTI and ETI (Chezem et al., 2017; Kim et al., 2020). SCHENGEN- and immune-lignin are also similarly rich in H-monolignols (Reyt et al., 2021). Together, this raises the intriguing possibility that the SCHENGEN pathway could be a neofunctionalization of the immune pathway, incorporating stress response into the endodermal developmental program. Whether induction of the immune response could be sufficient to replace the SCHENGEN pathway is an interesting question that can now be addressed using localized and targeted induction of the MTI pathway through FLS2 ectopic expression (Emonet et al., 2021).

## Notes

A.E and A.H. wrote the article and A.E. generated figures.

The author responsible for distribution of materials integral to the findings presented in this article in accordance with the policy described in the Instructions for Authors (https://academic.oup.com/plphys/pages/general-instructions) is: Angela Hay (hay@mpipz.mpg.de).
